# Optimising efficacy of antibiotics against systemic infection by varying dosage quantities and times

**DOI:** 10.1371/journal.pcbi.1008037

**Published:** 2020-08-03

**Authors:** Andy Hoyle, David Cairns, Iona Paterson, Stuart McMillan, Gabriela Ochoa, Andrew P. Desbois

**Affiliations:** 1 Computing Science and Mathematics, University of Stirling, Stirling, United Kingdom; 2 Institute of Aquaculture, University of Stirling, Stirling, United Kingdom; Emory University, UNITED STATES

## Abstract

Mass production and use of antibiotics has led to the rise of resistant bacteria, a problem possibly exacerbated by inappropriate and non-optimal application. Antibiotic treatment often follows *fixed-dose* regimens, with a standard dose of antibiotic administered equally spaced in time. But are such fixed-dose regimens optimal or can alternative regimens be designed to increase efficacy? Yet, few mathematical models have aimed to identify optimal treatments based on biological data of infections inside a living host. In addition, assumptions to make the mathematical models analytically tractable limit the search space of possible treatment regimens (e.g. to fixed-dose treatments). Here, we aimed to address these limitations by using experiments in a *Galleria mellonella* (insect) model of bacterial infection to create a fully parametrised mathematical model of a systemic *Vibrio* infection. We successfully validated this model with biological experiments, including treatments unseen by the mathematical model. Then, by applying artificial intelligence, this model was used to determine optimal antibiotic dosage regimens to treat the host to maximise survival while minimising total antibiotic used. As expected, host survival increased as total quantity of antibiotic applied during the course of treatment increased. However, many of the optimal regimens tended to follow a large initial ‘loading’ dose followed by doses of incremental reductions in antibiotic quantity (dose ‘tapering’). Moreover, application of the entire antibiotic in a single dose at the start of treatment was never optimal, except when the total quantity of antibiotic was very low. Importantly, the range of optimal regimens identified was broad enough to allow the antibiotic prescriber to choose a regimen based on additional criteria or preferences. Our findings demonstrate the utility of an insect host to model antibiotic therapies *in vivo* and the approach lays a foundation for future regimen optimisation for patient and societal benefits.

## Author summary

Research into optimal antibiotic use to improve efficacy is far behind that of cancer care, where personalised treatment is common. The integration of mathematical models with biological observations offers hope to optimise antibiotic use, and in this present study an *in vivo* insect model of systemic *Vibrio* infection was used for the first time to determine critical model parameters for optimal antibiotic treatment. By this approach, the optimal regimens tended to result from a large initial ‘loading’ dose followed by subsequent doses of incremental reductions in antibiotic quantity (dose ‘tapering’). The approach and findings of this study opens a new avenue towards optimal application of our precious antibiotic arsenal and may lead to more effective treatment regimens for patients, thus reducing the health and economic burdens associated with bacterial infections. Importantly, it can be argued that until we understand how to use a single antibiotic optimally, it is unlikely we will identify optimal ways to use multiple antibiotics simultaneously.

## Introduction

The increased availability of antibiotics has led to the overuse, and often inappropriate use, of these substances. This has resulted in bacterial diseases such as gonorrhoea, sepsis and tuberculosis becoming increasingly difficult to treat due to the emergence of multi-drug resistant strains [1–5]. Resistant bacteria pose significant health and economic burdens that has necessitated research into preventing their spread in attempts to prolong antibiotic effectiveness. Unfortunately, research indicates that the fight against antibiotic resistance will not be won by simply restricting *when* antibiotics are prescribed, therefore we must consider *how* they are prescribed [6–8].

Bacterial antibiotic resistance is not only of great concern for human patients, but it also has a significant impact in agriculture [9], aquaculture [10–11], horticulture [12], and the natural environment [13]. With the growth of the human population and the increased demand for animal protein in particular, the use of antibiotics in food production continues to increase [14]. Antibiotics are used extensively in these industries to treat infections and prevent diseases. Due to the importance of antibiotics for human and animal health, many countries have tight legislation surrounding the use of antibiotics within livestock production [15]; however, enforcement of such legislation still represents a major challenge in some territories.

The ‘prudent’ use of antibiotics has long been recommended as a way to slow the spread of antibiotic resistance [16]. However, for this to be fully effective, the treatment regimens under which they are administered should be optimal. Optimal antibiotic treatment strategies using a single antibiotic consist primarily of two variables: the dose and the duration of treatment. For most antibiotics, the drug developer identifies a treatment regimen which then is implemented by clinicians and veterinary surgeons when prescribing these antibiotics [17]. Conventional treatment regimens usually consist of a *fixed dose* administered typically equally split in time for a specified duration, e.g. 100 mg (or one tablet) given once per day for 7 days. Pharmacokinetics and pharmacodynamics studies of target populations are used to determine the dose and duration for these treatment regimens. However, one limitation of this approach is that it only provides information for the regimen being analysed and offers no indication for other potential regimens [18]. While these fixed-dose treatment regimens may be effective, they may not be the optimal dose or duration at which to administer the antibiotic most efficaciously or to prevent the emergence and spread of resistance [19].

The use of mathematical modelling in disease modelling and antibiotic resistance research has grown considerably over the past few decades and is now an invaluable tool [20]. Despite this, there is still little consensus on how individual antibiotic dosage regimens should be applied. D’Agata et al. [21] examined a series of models that included an immune response and a constant concentration of antibiotic (when present). These authors concluded that one of the most important factors was the early initiation of antibiotic treatment; however, they also argued that shorter antibiotic treatment durations resulted in the survival and selection of resistant strains [21]. Geli et al. [22] incorporated pharmacodynamics into their mathematical model by considering the antibiotic-induced death rate to be a function of the concentration of antibiotic present, although the concentration was modelled as a (non-dynamic) step function. These authors found that all antibiotic use increased the selection of resistance, regardless of the treatment regimen, although this was minimised for shorter durations of treatment, which also saw the time with symptoms decrease [22]. Ankomah and Levin [23] addressed the issue of a constant concentration of antibiotic by assuming that when antibiotics were not added to the system the concentration of antibiotic declined exponentially. They showed that under most conditions, high dose therapy is more effective than more moderate dosing to clear the infection and decrease the likelihood of emergence of antibiotic resistance; although these authors acknowledge that antibiotics can produce unwanted side-effects at greater concentrations [23]. Gjini and Brito [24] investigated the concept of adaptive treatments, whereby treatments are linked to bacterial load, further demonstrating that classical treatments (fixed dose and duration) are sub-optimal [24].

Given the increasing number of studies aiming to optimise antibiotic dosage regimens, advanced computational search algorithms, such as Genetic Algorithms (GA) [25], are significantly under-utilised in this field. This is in contrast to areas such as cancer chemotherapy where such approaches have been used for more than a decade [26–27]. GAs, which are a form of Evolutionary algorithms, allow a much wider search space to be studied and allows the relaxation of assumptions such as constant concentration of antibiotics or fixed daily doses. They are also adept for studying multi-objective optimisation problems, where the quality of a solution is defined by its performance in relation to several, often conflicting, objectives. Most real-world optimisation problems are multi-objective. However, they are traditionally transformed into a single-objective function, by means of a weighted sum of sub-functions, in order to make optimisation tractable. This approach suffers from a number of drawbacks; it assumes that we can capture preferences (weights), even before knowing the possible range of feasible solutions; and that these preferences remain static. Evolutionary algorithms have proven successful in finding high-quality solutions in high-dimensional spaces with difficult features such as constraints and discontinuities, and are currently the state of the art in many multi-objective optimisation problems [28].

Paterson et al. [29] was first to apply a GA to antibiotic dosing, allowing the size of each individual dose within a treatment regimen to vary. These authors found that tapering the antibiotic dosages, with a high first dose followed by subsequent decreasing doses, maximised the survival of hosts. Khan and Imran [30] confirmed these findings in a similar model, taking an optimal control theory approach. A second study using a GA [31] investigated a model of granulomas in a *Mycobacterium tuberculosis* infection, identifying the dose size and the dosing frequency to eradicate the bacteria quickly while keeping individual antibiotic dosages low.

Many of these previous studies are built around theoretical systems, with arbitrarily created parameter sets. There is little to no evidence at present whether these proposed treatment regimens will remain optimal inside an infected living host. Furthermore, there are very few mathematical models of antibiotic treatment systems that have been parameterised using biological data, and even the majority of these rely on *in vitro* studies [32–35].

There are two primary aims for this present study: firstly, to create and parameterise a mathematical model using host survival data from biological experiments in an infected living organism that can be treated with antibiotics; this mathematical model will be tested and validated by a set of follow up biological experiments. Secondly, the GA will be applied to this validated model to derive optimal treatment regimens, initially with the objective of maximising host survival and, subsequently, maximising host survival while minimising the total amount of antibiotic used. Due to the novel approach, which combines *in vivo* experiments with mathematical modelling and artificial intelligence, we consider only a scenario encompassing a single antibiotic used against only a single strain of pathogen, and the inclusion of multiple strains of varying susceptibility to the antibiotic is reserved for future study.

Animal models such as mice and rats are used in infection and treatment studies typically as a surrogate for humans, but despite the importance of such *in vivo* experiments there is a strong movement to reduce the number of vertebrates used in experimentation. Thus, less sentient alternative hosts such as insects are used increasingly for *in vivo* studies due to their greater ethical acceptance and low cost. In particular, the larva of the greater wax moth *Galleria mellonella* has become a popular choice amongst infection researchers and it has also been used successfully to assess the efficacy of antibiotic therapy *in vivo* [36–37]. Therefore, this insect host offers the ability to assess the *in vivo* efficacy of different antibiotic regimens against systemic bacterial infections. In earlier work, *G*. *mellonella* was demonstrated to be a suitable alternative host for studying the virulence of *Vibrio anguillarum*, an opportunistic bacterial pathogen of fish that causes sepsis in the host [38]. Virulent isolates of *V*. *anguillarum* can replicate inside the insect but antibiotics to which the bacterium is susceptible can be administered to save the host from a lethal inoculum of bacteria [38]. Notably, *V*. *anguillarum* was recently reported to be responsible for a lethal human infection [39]. Therefore, this host‒pathogen system was selected for application in the present study as a model of systemic *Vibrio* infection.

## Methods

### Biological experiments

#### Reagents, culture media and antibiotics

All chemicals and reagents were purchased from Sigma-Aldrich Ltd (Gillingham, UK) unless stated. All solutions were made with distilled water. Phosphate-buffered saline (PBS) was prepared according to Desbois and Coote [36]. Bacteria were cultured routinely on 1.5% (w/v) NaCl-supplemented tryptone soy agar (TSA; Oxoid, Basingstoke, UK) and broth (TSB; Oxoid), while Mueller-Hinton broth (MHB; Oxoid) supplemented with 2% (w/v) NaCl was used for minimum inhibitory concentration (MIC) determinations. Water, PBS and culture media were sterilised by autoclaving at 121°C for 15 min. Tetracycline hydrochloride (TET) was dissolved in distilled water, filter-sterilised (0.22 μ polyethersulfone; Millipore, Watford, UK) and then diluted to required concentration in PBS. Fresh stocks of TET were prepared daily.

#### Bacteria

*V*. *anguillarum* serotype O1 isolate Vib 79 (LMG 12101) [40] was kept routinely at -70°C in 15% (v/v) glycerol. Before use, bacteria were recovered initially onto agar, incubated at 22°C for 48 h, and then single colonies inoculated into broth. Cultures were incubated (22°C; 150 rpm; 12 h) until mid- to late-exponential phase and then bacterial cells were harvested by centrifugation (2700 × *g*; 15 min; 4°C). The cell pellet was washed by resuspension in PBS, centrifuged as before, re-suspended again in PBS, and then cell density determined by measuring absorbance at 600 nm (A_600_). Bacterial suspensions were diluted with PBS to 1×10^7^ CFU/mL, and all inoculums were serially diluted in PBS in quadruplicate and plated on TSA to confirm cell density.

#### Insects

*G*. *mellonella* larvae in their final instar stage were purchased (approximately 220 mg each; UK Waxworms Ltd, Sheffield, UK), stored in the dark at 4°C, and used within 14 days. A 50-μL Hamilton syringe (Sigma-Aldrich Ltd) was used for all injections of bacterial suspension, TET solution or PBS.

#### In vitro *minimum inhibitory concentration*

To identify a suitable TET dose to administer to infected *G*. *mellonella* larvae in an attempt to rescue them from a lethal inoculum of *V*. *anguillarum*, minimum inhibitory concentrations were determined according to a method modified from the Clinical and Laboratory Standards Institute standard [CLSI; 41]. Briefly, the wells in the last column of a flat-bottomed polystyrene 96-well microtitre plate (Sarstedt, Nümbrecht, Germany) were dispensed with 100 μL MHB containing antibiotic at double the greatest desired concentration for the assay. Two-fold dilutions were performed across the plate in fresh MHB and the final column contained just 100 μL MHB (no antibiotic control). Then, 5 μL of *V*. *anguillarum* suspension at 1×10^7^ CFU/mL was added to each well of duplicate rows on the plate. Microtitre plates were incubated (22°C; 180 rpm; 24h) and then the wells were examined by eye for growth. The MIC was recorded to be the lowest concentration of antibiotic at which no turbidity is observed.

#### Antibiotic treatment experiments

All experiments were completed in triplicate using larvae from different batches. Initial antibiotic experiments used groups containing 15 larvae (total n = 45) while model validation experiments used groups of 30 larvae (total n = 90). First, 10 μL of bacterial suspension was injected into larvae via the last left proleg before treatment at 2 h, 24 h and 48 h with 10 μL of TET solution, diluted to various concentrations in PBS, according to a published protocol [36]. The syringe was cleaned between experiments and treatment groups with consecutive washes of 1% (w/v) sodium hypochlorite, 70% ethanol and sterile water. A positive control group was injected with bacterial suspension, and PBS only instead of antibiotic. Three negative control groups were always prepared: one group that underwent no manipulation to control for background larval mortality (no manipulation control), a second group (uninfected control) that was injected with PBS only at initial challenge and all treatment time points, and a third group which assessed for the toxicity of the TET treatment by inoculation with the greatest antibiotic concentration used at each time point. There was never more than one death per control group per experiment. Larvae were stored in Petri dishes in the dark at 15°C for up to 192 h. Larvae were inspected every 24 h so that percentage survival could be calculated for each group; larvae were considered dead if they did not move after being touched with a sterile inoculation loop. This process was further refined for the model validation experiments when larvae were examined for movement under an Olympus VMZ 1× 4× VM stereo microscope (Tokyo, Japan).

#### Half-life of tetracycline

Inhibition of *V*. *anguillarum* growth by wax moth haemolymph spiked with TET was examined *in vitro* by disk diffusion assay to determine the relationship between TET concentration in haemolymph and the diameter of a growth inhibition zone. A single colony of *V*. *anguillarum* was added to 2 mL of PBS and vortexed for 30 s to suspend the bacteria. Bacterial lawns were prepared on 1.5% (w/v) NaCl-supplemented TSA plates by spreading 50 μL of bacterial suspension across the agar surface with a sterile cotton wool swab before drying for 1 h at room temperature. The haemolymph (ca. 5–20 μL from each animal) from 15 unmanipulated larvae was harvested according to McMillan et al. [38] and pooled in a bijou bottle on ice. Aliquots of haemolymph were prepared on ice to contain concentrations of TET between 0.625 mg/L and 40 mg/L, as described in MIC method (above). Then, 20 μL of each TET dilution was pipetted onto separate sterile antibiotic assay disks (Whatman 6 mm; GE Healthcare Life Sciences, Little Chalfont, UK). Once dry, the disks were placed onto the agar plates that had been inoculated with *V*. *anguillarum*, and then incubated (22°C; 24h). Then the diameters of the zones of inhibition were measured with calipers. This experiment was completed in triplicate. To estimate the decay rate of TET *in vivo*, half-life experiments were completed in wax moth larvae. TET was inoculated into wax moth larvae at 5 mg/kg. Haemolymph was harvested from larvae at 0.25 h, 0.5 h, 1 h, 1.5 h, 2 h, 4 h, 6 h, 8 h, 24 h and 30 h, then transferred onto antibiotic assay discs and tested for growth inhibition as described above. This experiment was performed in triplicate and larvae injected with PBS only were included as a negative control.

### Mathematical modelling

From here on in, for simplicity, we refer to *G*. *mellonella* larvae as the ‘host’, *V*. *anguillarum* as the ‘bacteria’ and TET as the ‘antibiotic’.

#### Bacteria

We assumed the bacteria population, with density given by *B*(*t*), is identical in terms of its antibiotic sensitivity – by which we mean the concentration of antibiotic required to kill off the population. The two actions in this model were: (i) replication, creating new bacterial cells, with rate *R*_+_, increasing the bacterial population; and (ii) the death of bacterial cells, *R*_−_, due to either the host immune system or by the antibiotic, reducing the bacterial population. For the bacterial population, the replication process was modelled by an exponential growth term (we initially tried a logistic growth function, but upon initial parameterising the model the carrying capacity was estimated to be 10^12^, which is considerably in excess of the bacterial load of 10^9^ at which the host dies [38]). The death rate due to the immune system was modelled by a saturating function, and the death rate due to the antibiotic was modelled by a sigmoidal function, where *A* is the quantity of antibiotic present to act against the bacterium causing the infection [28, 33–34]. The functional forms of these are given below in Eq (1):

$$R_{+}=\overset{Replication}{\overset{︷}{rB}}\;R_{-}=\underset{Immune}{\underset{︸}{mB^{n}}}+\underset{\mathrm{Antibiotic}\;induced}{\underset{︸}{\frac{a_{1}BA^{k}}{A^{k}+a_{2}^{k}}}}$$ (1)

The number of bacteria was modelled using a Markov chain approach, specifically the Gillespie Algorithm [42] (described below). Due to the high population size of bacteria, up to 10^9^, the standard Gillespie Algorithm would have a high run-time (as the time between each individual event would be close to zero). Hence, we took an approximation of the Gillespie Algorithm, known as Tau-leaping [43]. Following preliminary runs of the mathematical model, we settled on a fixed time step of *τ* = 0.25 (15 minutes), and updated the number of bacteria using Eq (2)

$$B\left(t+\tau \right)=B\left(t\right)+P\left(\tau R_{+}\left(t\right)\right)-P\left(\tau R_{-}\left(t\right)\right)$$ (2)

where *P*(*τx*(*t*)) is a Poisson distributed random variable with mean *τx*(*t*). Initially, the bacteria population was *B*(0) = 10^5^, matching the biological experiments. To model host heterogeneity, for each run of the mathematical model, *r* and *m* were drawn from a normal distribution with means *r*_*μ*_ and *m*_*μ*_ respectively, and a shared standard deviation *v*, i.e. *r*~*N*(*r*_*μ*_,*v*) and *m*~*N*(*m*_*μ*_,*v*).

#### Antibiotic

The antibiotic concentration was based on a standard pharmacokinetic / pharmacodynamics (PK/PD) approach, where *A* is the quantity of antibiotic present to act against the bacterium causing the infection, and *a* is the decay rate of the antibiotic. We modelled this using the commonly taken approach [44], with equations shown in (3):

A(t+τ)=A(t)+P(τaA(t)) (3)

As the time *t* passed a dosage time point, *t*_*i*_ = 2h, 24h, 48h, the next dose of antibiotic, *d*_*i*_, was applied to the system. Initially, we assumed there was no antibiotic in the host, so *A*(0) = 0.

As this was a stochastic model, the behaviour of the antibiotic and bacteria population size changed with each run. For this reason, we carried out 5000 runs of the model, and counted the number of runs where treatment was a ‘success’: a model run was a ‘success’ if the host survived to 192 h, as measured by the bacterial population size staying below a threshold, i.e. *B*(*t*)<*B*_*dead*_ for all *t*∈[0,192]; whereas a model run was a ‘failure’ if the host died, if the bacterial population exceeded the threshold, i.e. *B*(*t*)>*B*_*dead*_ for any *t*∈[0,192]. McMillan et al (2015) found in their experiments that the host (the larvae) died when the bacterial abundances reached approximately 10^9^ [38]; this provides a value for the ‘death threshold’ *B*_*dead*_ of 10^9^. The success of an antibiotic treatment was then measured by the fraction of runs where the host survived to 192 h, denoted *N*_*surv*_.

The parameter definitions are given in Table 1.

**Table 1:** Parameter definitions. The parameter values were those found during the optimisation process in the Results section.

Parameter	Definition	Value
*r*_*μ*_	Average Replication rate of bacteria	0.4779
*m*_*μ*_	Co-efficient for the host immune response	0.6772
*n*	Hill co-efficient in the immune response	0.9193
*v*	Standard deviation for host heterogeneity	0.0525
*a*_1_	Maximum kill rate of antibiotic	0.7281
*a*_2_	Level of antibiotic giving half max kill rate	0.1910
*k*	Hill co-efficient in AB induced death.	2.9821
*a*	Decay rate of antibiotic *(half-life = 5*.*9hrs)*	0.1174
*B*_*dead*_	Bacterial load at which the host dies [38]	10^9^

### Computational Optimisation

Genetic Algorithms (GA) were proposed by John Holland in the early 1970s [25]. They belong to the larger class of evolutionary algorithms, which generate solutions to optimisation problems using techniques inspired by natural evolution, such as inheritance, mutation, selection and crossover [45]. GAs have previously been used to generate treatment schedules for chemotherapy of cancer patients [26–27], but have rarely been used in antibiotic therapy [29,31]. Despite being a randomised search GAs are by no means random, instead they use historical information to direct the search into the region of better performance within the search space. Here, the GA was used for two purposes:

#### Parameterisation

To parametrise the mathematical model, we used the GA to find a set of parameters that allowed the model to best match the biological data. The GA generated sets of parameter values for *r*_*μ*_, *v*, *m*_*μ*_, *n*, *a*, *a*_1_, *a*_2_ and *k*, represented by a vector of real numbers. For each set, the GA ran the mathematical model 5000 times, and computed how well the mathematical model fitted the data, as measured by a least squares approach calculating the difference between host survival at each 24-h interval from the mathematical model and the biological experiments; the fit is denoted by the function *F*_*par*_ and is given in (5):

$$F_{par}=\underset{No\;\mathrm{Antibiotic}\;data}{\underset{︸}{\sum_{i=1}^{3}\sum_{T=0:}^{5}{\left(B(24T)-D_{i,24T}\right)}^{2}}}+\underset{\mathrm{Antibiotic}\;data}{\underset{︸}{\sum_{j=1}^{3}\sum_{T=0:}^{8}{\left(B(24T)-D_{j,24T}\right)}^{2}}}$$ (5)

The first term compared the model to the data in McMIllan et al. [38], summarised in the Supporting Information (S1 Table), where hosts were infected but not treated with antibiotic, and host survival determined at 24-h intervals from 0 h to 120 h. The second term compared the mathematical model to the biological data from Table 2, where hosts were infected and treated with antibiotic, and host survival measured at 24-h intervals from 0 h to 192 h.

**Table 2:** Host survival at the end of each 24-h interval for different ‘fixed dose’ treatments, i.e. 0.9 mg either administered as a single dose, or split equally over 2 days, or split equally over 3 days (n = 45). PBS = phosphate-buffered saline; Vib 79 = *V*. *anguillarum* (bacterium).

group	infected	treatment (mg/kg)			Host survival (proportion of host alive at each measure)							
	0 h	2 h	24 h	48 h	24h	48h	72h	96h	120h	144h	168h	192h
unmanipulated	-	-	-	-	1	1	1	1	1	1	1	1
PBS only	PBS	PBS	PBS	PBS	1	1	1	1	1	1	1	1
antibiotics only	PBS	0.9	0.45	0.3	1	1	1	1	1	1	1	1
positive control	Vib 79	PBS	PBS	PBS	1	0.02	0	0	0	0	0	0
0.9 mg	Vib 79	0.9	PBS	PBS	1	1.00	0.98	0.82	0.69	0.62	0.53	0.53
0.9 mg	Vib 79	0.45	0.45	PBS	1	1.00	0.89	0.78	0.67	0.58	0.42	0.38
0.9 mg	Vib 79	0.3	0.3	0.3	1	0.96	0.44	0.27	0.13	0.09	0.04	0.04

#### Optimising dosage regimens

Antibiotic regimens are represented by dosage vectors (*d*_1_, *d*_2_, *d*_3_,…), where *d*_*i*_ denotes the quantity of antibiotic (measured in mg) to be given at time *t*_*i*_. The GA aimed to find the optimal dosage vector that minimised our objective function, which comprised of up to two components: first, to maximise the number of runs of the mathematical model where the host survived the infection, i.e. *B*(*t*)<*B*_*dead*_ for all *t*, denoted *N*_*surv*_; second, to minimise the total amount of antibiotic used, as measured by the sum of the entries in the dosage vector, ∑_*i*_*d*_*i*_. In addition, we had constraints: ∑_*i*_*d*_*i*_≤0.9 mg, where 0≤*d*_*i*_≤0.9 (except for the final section in the results, ‘(iv) Maximise survival vs. minimise total antibiotic’, where we increased the total quantity of antibiotic). For each regimen, the GA ran the mathematical model 5000 times, with a population size of 50 potential solutions (dosage vectors), for 80 generations and the whole process was repeated 50 times. At the end of the complete GA run, we produced 50 sets (one from each GA run), each containing 50 possible solutions (dosage vectors). The run lengths, generations and population sizes were arrived at from prior calibration of the configuration that confirmed that minimal further improvement in performance was gained by increasing these values.

## Results

### Initial antibiotic experiments

Initially the larvae hosts were treated with 0.9 mg of tetracycline, split into fixed dosages at 24-h intervals, either: all 0.9 mg in one dose at 2 h; split into two equal doses of 0.45 mg each at 2 h and 24 h; split into three equal doses of 0.3 mg each at 2 h, 24 h and 48 h. The survival rates of the larvae are shown in Table 2.

#### Half-life experiments

**Fig 1:**
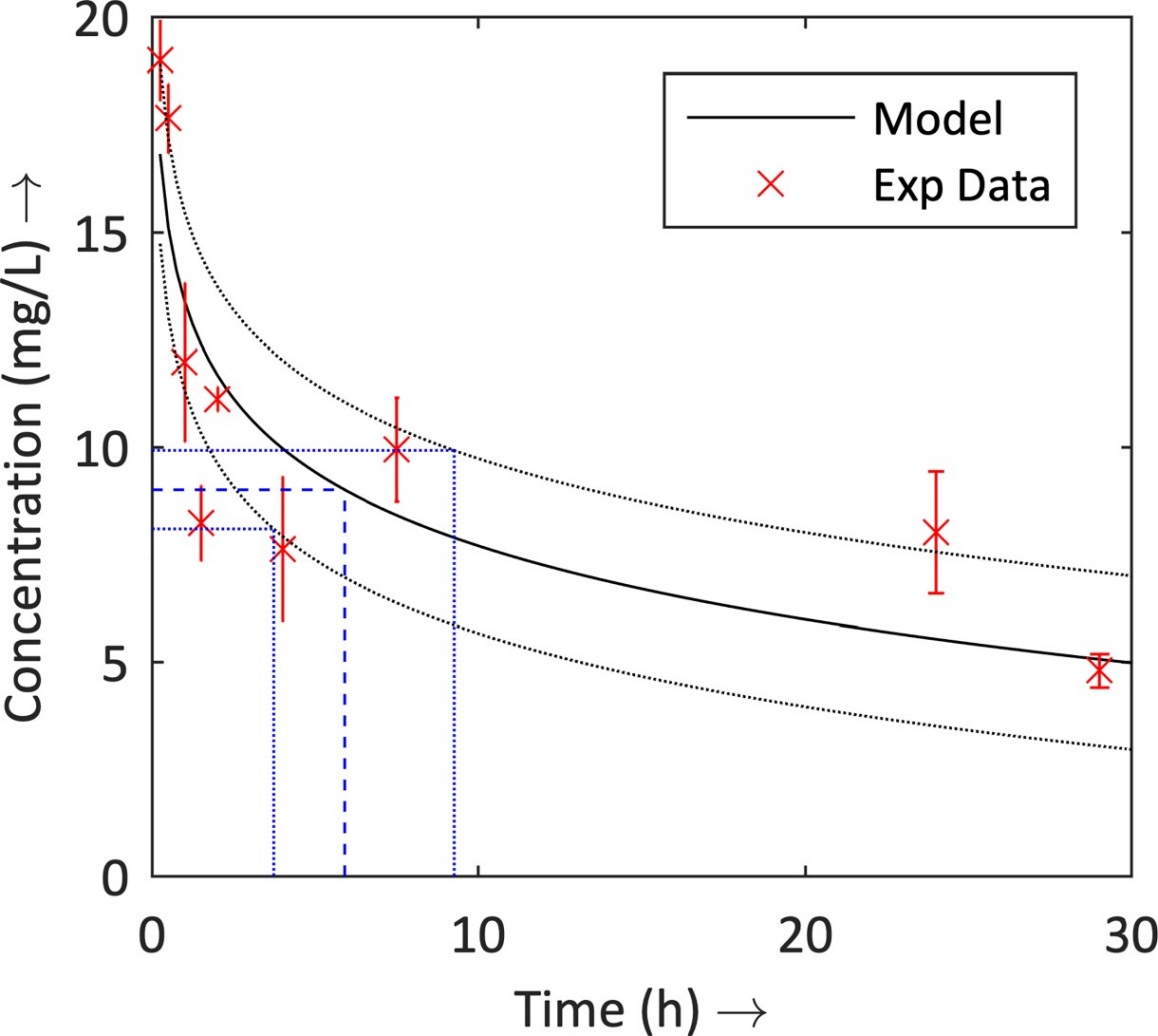
Decay rate of the antibiotic (TET) over time in *G*. *mellonella* host. Biological data of the decay rate of the antibiotic over time in *G*. *mellonella* (red points); and fitted curves for the experimental data (black lines) and the estimate for the half-life of the antibiotic (blue lines). The black and blue dotted lines represent the respective 95% confidence intervals.

To gain a more accurate estimate for the decay rate of the antibiotic (TET), the antibiotic activity in the haemolymph was measured over time (Fig 1, with exact values in Supporting Information (S2 Table)). Fitting a curve to these data, along with the 95% confidence intervals, provided an estimation of the half-life of TET to be approximately 5.89 h, with a confidence interval of 3.72–9.26 h, which is in accordance with previous biological estimates [46]. This provided an appropriate interval for the decay rate of the antibiotic, *a*. (Following subsequent application of the GA, a half-life of 5.90 h was settled upon.)

### Parameterisation of the mathematical model

**Fig 2:**
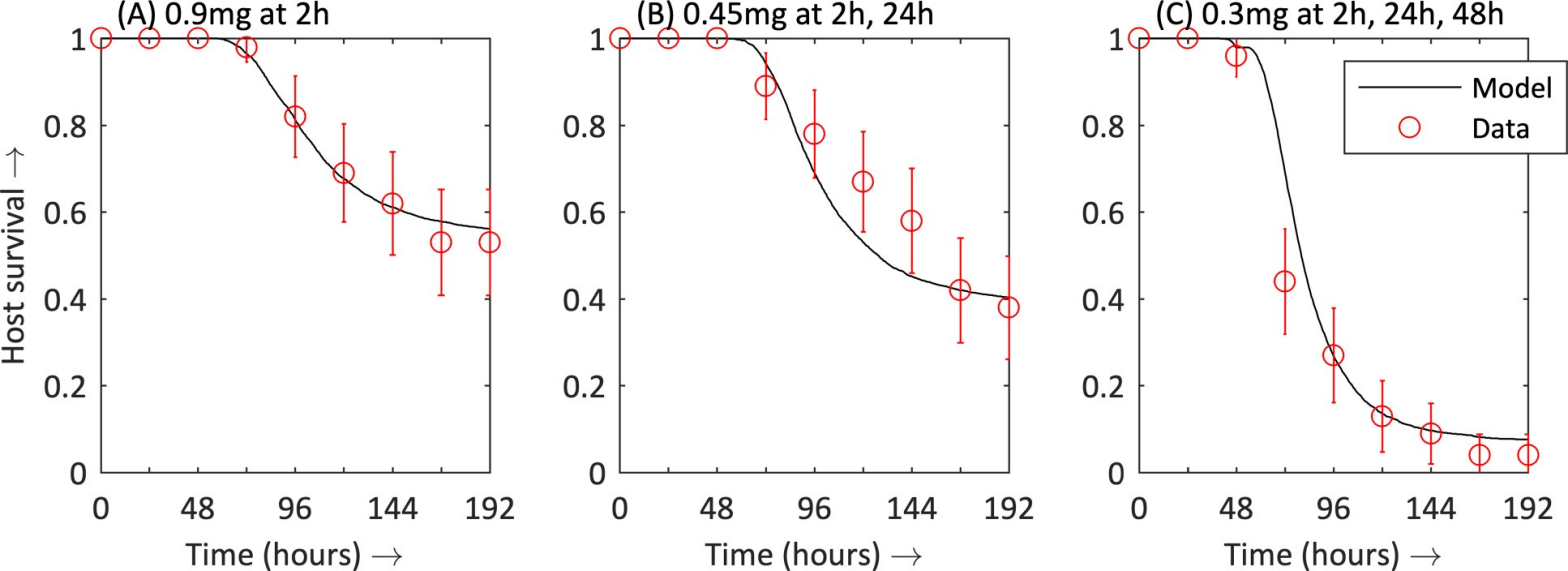
Comparison of mathematical model and experimental results for survival rates for different ‘fixed dose’ treatments, with 0.9 mg either administered as a single dose, or split equally over 2 days, or split equally over 3 days: (A) 0.9 mg administered at 2 h; (B) 0.45 mg administered at 2 h and at 24 h; (C) 0.3 mg administered at 2 h, 24 h and 48 h. n = 45 for biological experiments; n = 5000 for mathematical model.

The survival rates in Table 2, and the survival rates from McMillan et al. [38] summarised in Supporting Information (S1 Table), were used to parameterize *r*_*μ*_, *v*, *m*_*μ*_, *n a*, *a*_1_, *a*_2_ and *k*. The GA showed strong convergence and provided a set of parameter values that best fit the data. The model with these parameters showed a reasonable fit with the biological data (Table 2) when mean host survival over time was plotted (Fig 2). (In the Supporting Information (S1 Fig), the bacteria densities within the host are plotted over time for case the infected host is not treated with antibiotics. Comparing the model output with the biological data from [38] shows that the model’s maximum growth rate of bacteria is biologically credible).

**Table 3:** Host survival from the biological validation experiments. Host survival was recorded at 192 h for five different antibiotic treatment regimens, with both the ‘raw’ data given, along with the ‘normalised’ data, whereby host survival for four of the treatments were increased by 0.28 to bring host survival for (0.45,0.45) and (0.9,0) treatments in line with those in the initial experiments, in Table 2. (n = 90.) Full data in the Supporting Information (S3 Table).

infected	treatment (mg/kg)		Host survival at 192h	
0 h	2h	24h	Raw	Normalised
Vib 79	0.20	0.70	0	--
Vib 79	0.45	0.45	0.10	0.38
Vib 79	0.56	0.34	0.21	0.49
Vib 79	0.76	0.14	0.26	0.54
Vib 79	0.9	PBS	0.24	0.52

### Validating the Model

**Fig 3:**
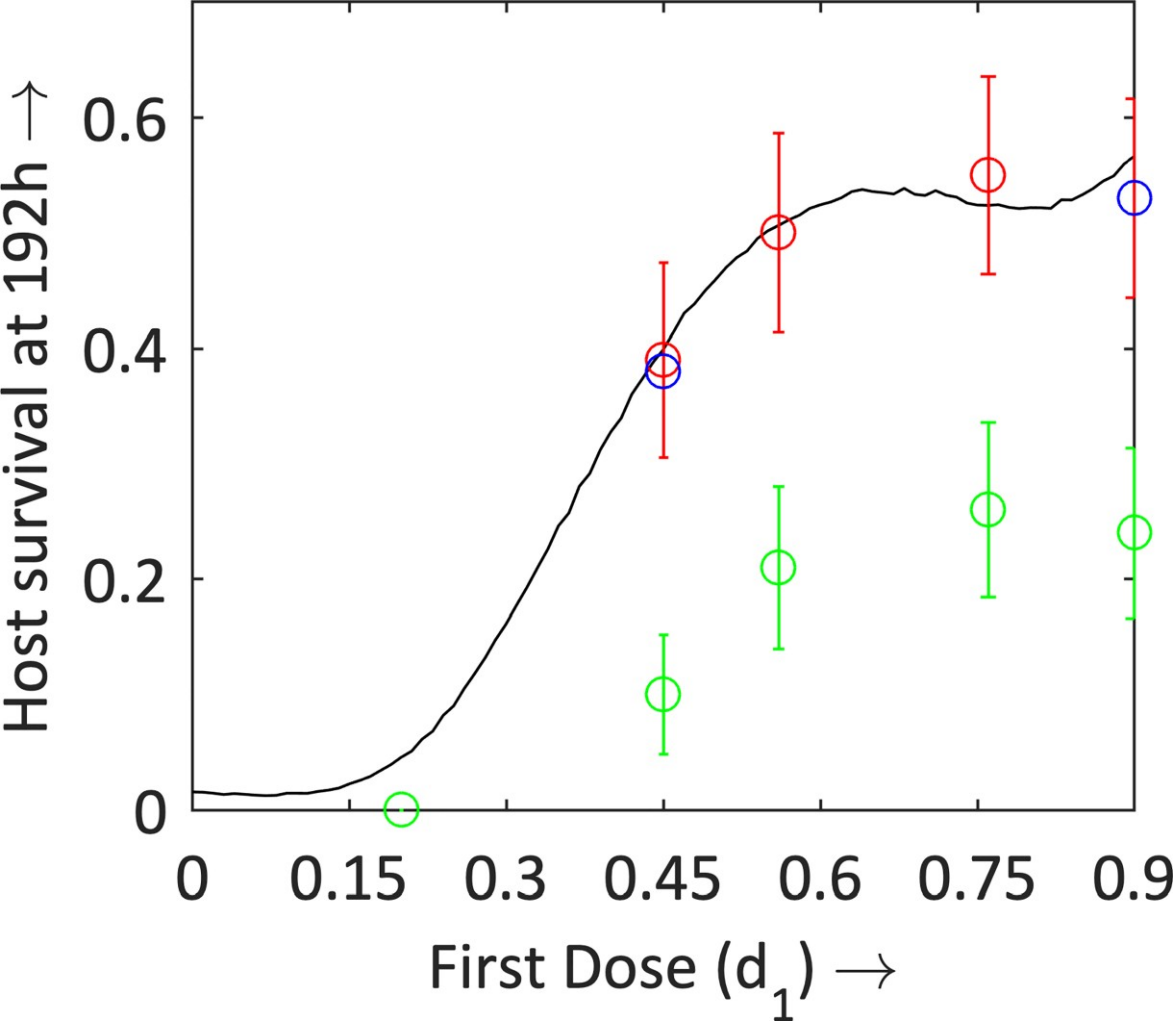
Survival rates at 192 h for various first doses, *d*_1_; where the second dose is (0.9−*d*_1_). The mathematical model results are in black; blue circles represent the initial biological experimental data (Table 2), and green circles are the additional biological experiments (Table 3). Red circles represent the *normalised* data from Table 3 (with each of the four points increased by 0.28). (The model runs for these solutions were increased to 10000 to confirm the accuracy of the results).

To determine the effect of the size of the first dose, and whether a bigger first dose leads to a higher survival, we consider a general two-dose strategy of the form (*d*_1_, 0.9−*d*_1_), with dose *d*_1_ at 2 h and the second (remaining) dose at 24 h. This strategy used 0.9 mg of antibiotic in total. Our parameterised mathematical model was run for values of *d*_1_ from 0 to 0.9, in increments of 0.01, with survival rate recorded at 192 h (Fig 3). There is a strong correlation between the first dose and host survival, with a Spearman Rank co-efficient of 0.96. There is a small drop in host survival for first doses *d*_1_ between 0.65 and 0.85, however, this is only small.

To validate the mathematical model, five two-dose treatments were then chosen, and biological experiments of these treatments were carried out for n = 90 larvae (Table 3; the full data set, with survival at the end of each 24-h interval is given in the Supporting Information (S3 Table)).

The survival rates in these *in vivo* experiments (Table 3) were significantly lower than those of the initial experiments (Table 2), and a direct comparison of the regimens (0.9,0) and (0.45,0.45) showed a consistent drop of ~0.28 in the survival rate at 192 h. This is likely due to seasonal variation in the condition of the larvae, which can occur [47–48]. Therefore, for modelling purposes, we normalised the survival rate of the regimens (0.45,0.45), (0.56,0.34), (0.76,0.14) and (0.9,0) by increasing the survival rates universally at 192 h by 0.28. Comparing these new scaled survival rates to our mathematical model (Fig 3) showed that the model successfully predicted the survival rates of the previously untested regimens.

### Optimisation of treatment regimens

**Fig 4:**
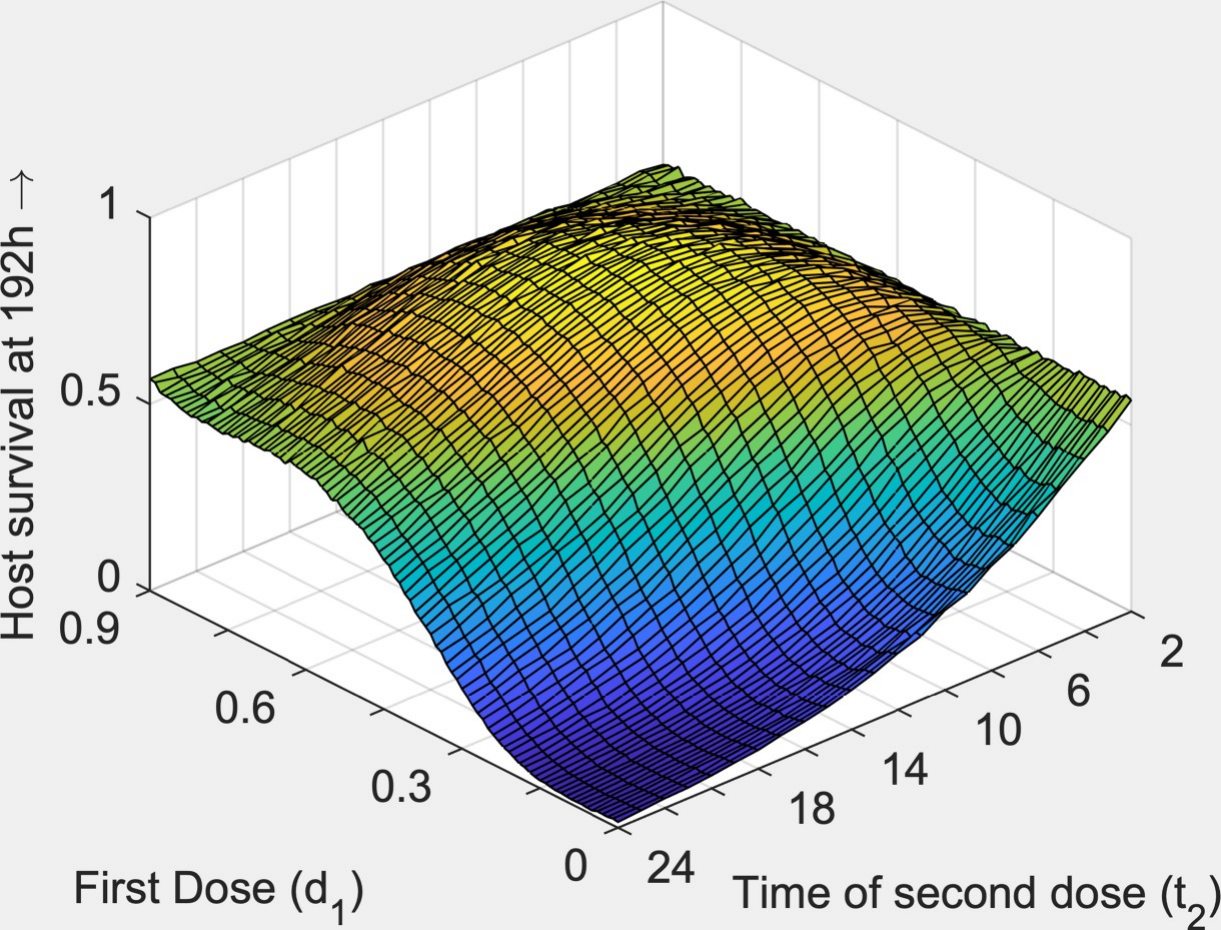
Two-dose treatment regimen with the first dose *d*_*1*_ is taken at 2 h and the second dose 0.9-*d*_*1*_ at *t*_*2*_ h. Host survival at 192 h is plotted against the size of the first dose *d*_*1*_: and the time of the second dose *t*_*2*_. Model runs = 10000.

**Fig 5:**
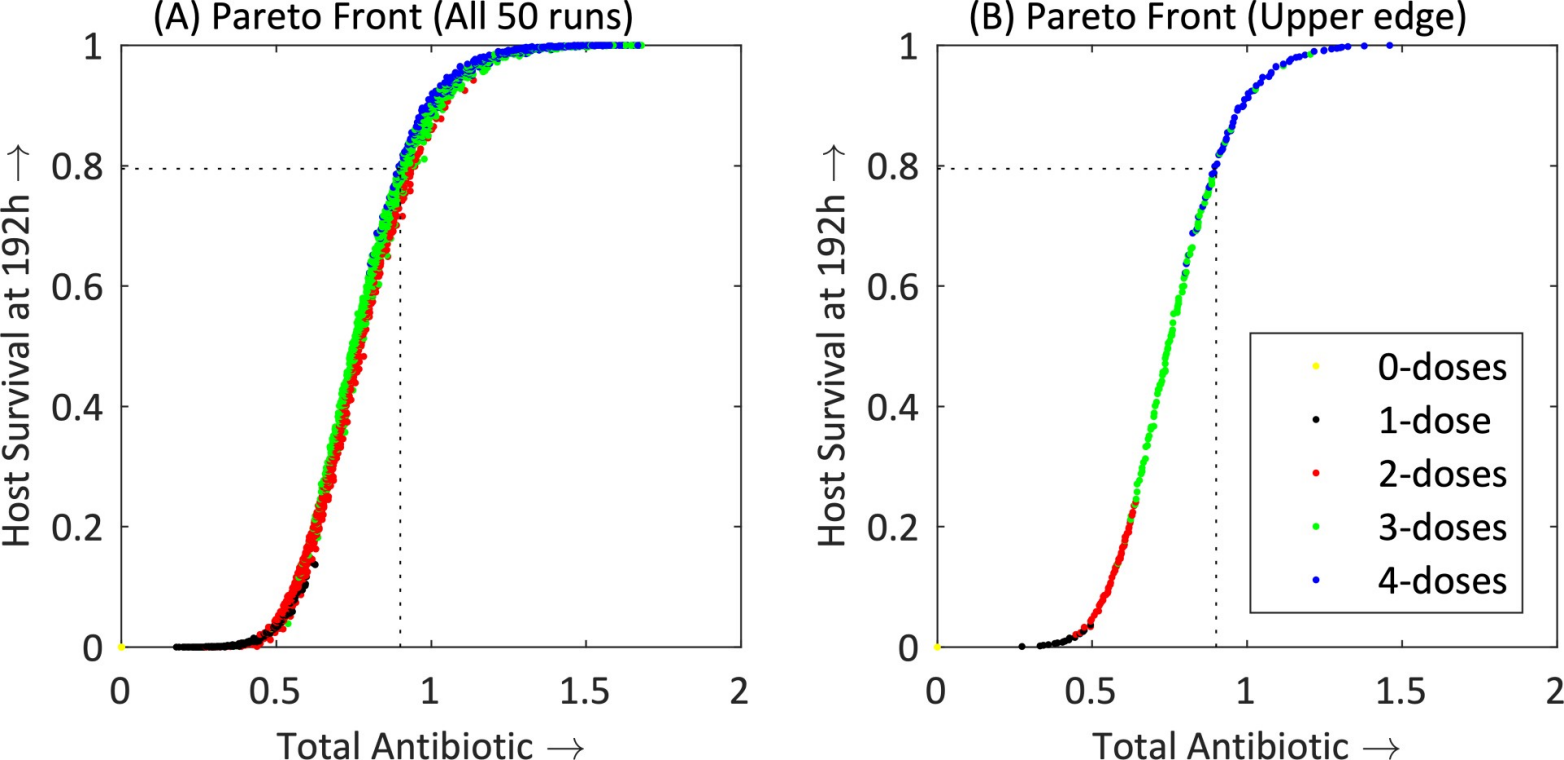
(A) Combined Pareto Fronts for 50 repeat runs; (B) subset of points along the upper edge of combined Pareto Fronts in (A). Both graphs show the trade-off between the total amount of antibiotic used in a treatment regimen and maximum host survival at 192 h. The colours of the points represent the number of (non-zero) antibiotic doses used to achieve that optimal point. Population = 50, generations = 80, model runs = 5000, repetitions = 50.

We applied the GA to the parameterised mathematical model to search for optimal antibiotic dosage regimens to maximise host survival at 192 h under various conditions:

i ***Daily dose treatments*:** Taking a daily dose treatment strategy, with doses at 2 h, 24 h, 48 h and 72 h, we found that to maximise host survival at 192 h, using 0.9 mg of TET, the best strategy was to administer all 0.9 mg at 2 h, with 0 mg at the remaining dose times. This gave a host survival rate at 192h of 0.566.
ii ***Two-dose*, *variable timings*:** Next, we allowed the timing of the doses to vary, initially limiting the solutions to two-dose treatments, whereby a dose *d*_1_ is given at 2 h, and a second (remaining) dose of 0.9-*d*_1_ is given at *t*_2_ hours. Given we only had two variables, *d*_1_ and *t*_2_, we carried out a brute force (exhaustive) search for *d*_1_ between 0 and 0.9 (in intervals of 0.01), and *t*_2_ at hourly intervals between 3 h and 24 h. In Fig 4, we plot host survival at 192 h against the time of the second dose *t*_2_, and the size of the first dose *d*_1_. We found that the optimal strategy was to administer a first dose of 0.54 mg of TET at 2 h and a second dose of 0.36 mg at 11 h, giving a survival rate at 192 h of 0.795. This is significantly greater than the survival rate of 0.566 for administering all 0.9 mg of TET in a single dose at 2 h.

iii ***Multiple-doses*, *variable timings*:** We extended the search to find the optimal treatment regimen, using 0.9 mg of antibiotic, by allowing any number of doses. A GA was applied to this problem, allowing the individual dosage quantities and timings of these doses to vary, to find the regimen that maximised host survival at 192 h. We found the optimal regimen was a four-dose strategy, giving: 0.43 mg at 2 h, 0.22 mg at 7.1 h, 0.13 mg at 11.7 h, and 0.13 mg at 16.8 h, which gave a host survival rate of 0.803. (Note, total antibiotic adds to 0.91 mg due to rounding.) Given this is a stochastic model, it would be difficult to test experimentally *in vivo* whether the improvement over the two-dose treatment above, with survival of 0.795, is significant.
iv ***Maximise survival vs*. *minimise total antibiotic***: Taking a multi-objective approach, we aimed to maximise host survival at 192 h while minimising the total quantity of antibiotic used. Again, we allowed both the individual doses and the timing of these doses to vary. In Fig 5, Pareto Front is plotted–in the context of multi-objective optimisation, a Pareto Front is a set of non-dominated solutions, which are considered optimal if no objective can be improved without sacrificing at least one other objective; this was done using a well-known GA suited for multiple objectives, NSGA-II [49]. Each point represents the host survival rate and quantity of antibiotic used, assuming that that the amount of antibiotic is used optimally. Before deriving solutions for a particular regimen, we had to ensure that the results were consistent and therefore we repeated the optimisation process over 50 independent runs. The combined set of Pareto Fronts for these 50 optimisation runs is given in Fig 5A and shows a consistency across the repeat runs with regard to the trade-off between total antibiotic use and host survival at 192 h. From this set of 50 repeat runs, we extracted the points along the upper edge of the combined Pareto Fronts to produce a final composite Pareto Front that is optimal across all 50 runs (Fig 5B). There exists a strong, non-linear, positive correlation between survival and the total quantity of antibiotic used. In fact, for total antibiotic dosage of between 0.56 mg and 0.95 mg, there is a very strong linear correlation (*r* = 0.997), with a gradient of 2.12, i.e. every 0.1 mg increase in antibiotic gives an increase in host survival at 192 h of 0.212. In addition, we saw almost distinct boundaries between where the optimal regimen involved increasing the number of doses: a single (initial) dose when using less than 0.49 mg, two-doses when using between 0.49 mg and 0.64 mg in total, three-doses when using between 0.64 mg and 0.9 mg in total, and four-doses when using more than 0.9 mg in total.

To understand the form of the optimal dosage vectors in the Pareto front, we explored the treatment regimens that were found by the GA and extracted the best regimen for a particular objective. Table 4 shows the solutions from Fig 5B where we consider treatments that have a survival rate at 192 h of at least (a) 0.9, and (b) 0.99 for different objectives. These objectives were: (i) the least total antibiotic used (i.e. to minimise ∑*d*_*i*_); (ii) the least number of doses (i.e. least number of non-zero entries in dosage vector); (iii) the lowest maximum dose (i.e. to minimise max(*d*_*i*_)); (iv) the earliest final dose (i.e. to minimise max(t_i_)).

**Table 4:** Optimal treatment regimens from within the Pareto Front (Fig 5B) for different criteria, given host survival at 192 h of at least (a) 0.9, or (b) 0.99. (*Only 4-dose treatments were found along the upper edge of Pareto Front with host survival more than 0.99).

Objective	Dose 1	Dose 2	Dose 3	Dose 4	Total AB	Survival at 192h
style="border-right:thick">Lowest total antibiotic	0.48 mg 2 h	0.28 mg 9.0 h	0.24 mg 17.1 h	--	1.00 mg	0.900
	0.52 mg 2 h	0.38 mg 8.4 h	0.15 mg 17.4 h	0.17 mg 21.1 h	1.22 mg	0.990
Least number of doses	0.50 mg 2 h	0.29 mg 7.6 h	0.23 mg 14.5 h	--	1.02 mg	0.927
	0.60 mg 2 h	0.34 mg 9.0 h	0.27 mg 14.3 h	--	1.21 mg	0.988*
Lowest maximum dose:	0.33 mg 2 h	0.23 mg 5.7 h	0.28 mg 11.1 h	0.18 mg 17.8 h	1.02 mg	0.925
	0.44 mg 2 h	0.29 mg 6.7 h	0.18 mg 10.9 h	0.34 mg 18.8 h	1.25 mg	0.992
Earliest final dose:	0.38 mg 2 h	0.22 mg 4.4 h	0.17 mg 9.7 h	0.24 mg 13.4 h	1.01 mg	0.913
	0.48 mg 2 h	0.33 mg 7.0 h	0.18 mg 12.2 h	0.31 mg 17.5 h	1.30 mg	0.997

All the treatments in Table 4 have a similar pattern in that the first dose is the largest dose, with many of the subsequent doses decreasing throughout, e.g. (0.48, 0.28, 0.24). There is also an appearance of possible additional trade-offs. For example, comparing row 1 and row 5 in Table 4, in reducing the maximum concentration of individual doses may require an increase in the total quantity of antibiotic used and an increase in the duration of treatment. Calculating the Spearman Rank co-efficient for Fig 5B, we get 0.55, indicating that there is no longer a strong correlation between the first dose and host survival; given the wider range of treatments in terms of number of doses (e.g. more, smaller doses) and timings (e.g. smaller doses closer together), this is to be expected.

## Discussion

The first aim of this study was to build and parameterise a mathematical model that accurately represents a systemic bacterial infection (*V*. *anguillarum*) in an *in vivo* host (*G*. *mellonella*) that can be treated with different fixed-dose antibiotic regimens. The mathematical model of this system was then tested with additional experiments, including some new antibiotic regimens where daily doses were no longer fixed. The model was shown to perform well in these subsequent experiments, and hence provides a useful new approach for researchers investigating the optimisation of antibiotic therapy.

The second aim of this study was to apply an advanced computational search technique, a Genetic Algorithm (GA), to this model to find optimal antibiotic dosage regimens that maximised host survival, while also minimising total antibiotic usage. In searching for the optimal regimen, we relaxed the commonly made assumptions of fixed doses (where each dose is, say, X mg) at fixed intervals (e.g. every 24 h) – the widened search space of possible treatments makes the application of artificial intelligence algorithms essential. When using a fixed total antibiotic dose of 0.9 mg, the best two-dose regimen was to apply 0.54 mg at 2 h and 0.36 mg at 11 h, giving a host survival rate of 0.795. This only increased slightly to 0.803 with a four-dose regimen. However, both of these were predicted to be significantly greater survival than administering all 0.9 mg in a single dose at 2 h. When aiming to minimise the total quantity of antibiotic used while maximising host survival, all the treatments found (including those in Table 4) show a similar pattern: the first dose is the largest dose, with many of the subsequent doses decreasing thereafter. Many of the treatment durations were relatively short, with the final dose being applied before the 24 h mark (Table 4). Here, the treatment intervals were relatively consistent; for example, with antibiotic being administered at 2 h, 9 h and 17 h (Table 4, row 1) or 2 h, 7 h, 12 h and 17 h (Table 4, row 8). Treatment intervals could therefore be an important, yet understudied area, with few studies of their effect on survival [50] or development of resistance [51]. There was also an appearance of possible additional trade-offs: for example, in reducing the maximum concentration of any individual doses required an increase in the total quantity of antibiotic used and an increase in treatment duration; similarly shorter dosage regimens appeared to increase the total antibiotic required and increased the individual dosage sizes. A further study exploring these trade-offs, carrying out a multi-objective optimisation approach with the four objectives in Table 4, along with maximising host survival, is certainly worthy of attention. Furthermore, the mathematical model was parameterised using a standard GA; however, when parameters are encoded as real numbers we can apply modern evolutionary algorithms that specialise in the continuous domain, such as Differential Evolution (DE) [52–53] and Covariance Matrix Adaptation Evolution Strategies (CMA-ES) [54–55]. It may be possible that these methods can produce improved solutions in shorter computational time, which would be an interesting follow-up study.

Significantly, this present study provides further evidence supporting the previous theoretical results that optimal dosage patterns for antibiotics follow a tapering pattern [29–30]. Given that the tapered treatment patterns in this present study were derived from a biologically validated model, it provides evidence that further research into this observation is needed. Obviously, the next step would be to validate these optimal treatments in follow-up biological experiments, directly testing conventional fixed-dose treatments against the optimal tapered treatments and evaluating host survival across time. Furthermore, this present study focused on the single outcome of host survival; however, future studies could integrate further beneficial outcomes such as the risk associated with selecting for antibiotic-resistant strains [56–58].

Our results also reinforce previous findings in humans, including studies that have shown that shorter treatment regimens can be effective in treating bacterial infections [59–60] and the use of initial high loading dose treatments being beneficial in treating patients in critical care medicine [61]. Interestingly, tapered regimens are effective when treating infections caused by *Clostridium difficile* [62–63]; however, the use of tapered regimens has resulted in sub-optimal performance in bacterial clearance in some previous infection studies [35,64] underlining the importance of deriving an *optimal* tapered strategy.

The duration under which a bacterium is exposed to antibiotics increases the likelihood of resistance developing [65]. The selection of current treatment durations is relatively arbitrary, albeit with supporting data from pharmacokinetic and pharmacodynamics trials, to ensure therapeutic concentrations of antibiotic are maintained in the host, and several studies have indicated that shorter treatment regimens can be just as effective [66–68]. By altering the interval between the constant doses, the GA produced treatment regimens that were shorter and showed little change in the total quantity of antibiotic used. Shorter treatment durations have been identified as being as effective as longer durations in treating a number of bacterial diseases [60,67,69], indicating that current treatment guidelines, while effective, may not be the optimal way to administer antibiotics. In addition, exposing the environment to larger quantities of antibiotic can increase the abundance of resistant bacteria [70–71]. Optimal antibiotic treatments may also be highly dependent on the current quantity of the target bacterial cells present in the host [72–76], and it would be interesting to see how well the optimal treatments would perform across populations of individuals where variation in drug metabolism would play a significant role.

The conventional treatment regimen of a standard dose administered at equal intervals in time is appealing to both manufacturers and patients. However, to increase the effectiveness of antibiotics may require a move away from these conventional regimens. Changing the interval between doses of antibiotics would be more preferable for manufacturers, as the doses of antibiotic remain constant and a single, standard product is manufactured. This shifts the burden of responsibility to adhere to the new regimes more to the prescriber and the patient. Altering the quantities of antibiotics given at set time intervals is another approach that could be effective, and in such a scenario different doses are prepared by the manufacturer for administration during the course of treatment. The constant time interval between doses is probably simpler for the patient to comply and, interestingly, patient compliance rates are greatest (almost 100%) for daily doses [77–78]. In the most complex scenario, both the quantities of antibiotic given and the timings of each treatment during therapy are altered for an optimal outcome. Unfortunately, lack of patient compliance remains a common problem [79–80] and much further work is needed before changes to conventional treatment regimens could be translated to the clinic. Of course, this would include the complete involvement of stakeholders and a full understanding and consent around acceptable levels of safety and risk, particularly the implications of inadvertently deviating from an optimised treatment. In this regard, it may be in veterinary medicine where significant gains may be achieved more quickly.

With the increase in antibiotic resistant bacteria, research has begun to examine the effectiveness of using multiple antibiotics, either sequentially or together in combinations [81–82]. Still, there is a need to ensure single antibiotics are used in an optimal manner, as this is another key approach that may deliver considerable benefit to patients. The findings of this present study highlight the potential amendments that could be made to single daily fixed-dose antibiotic treatment regimens to increase their efficacy, thus reducing the health and economic burdens associated with bacterial infections.

## Supporting information

S1 Table Host survival data from [38]. (DOCX)

S2 Table Experimental data from the tetracycline half-life experiments. (DOCX)

S3 Table Full experimental data for the validation biological experiments. (DOCX)

S1 Fig Plot of bacterial density in a host over time with no treatment. (EPS)
