# Supplementary material for: Optimising efficacy of antibiotics against systemic infection by varying dosage quantities and times
Source: PLoS Comput Biol. 2020 Aug 3;16(8):e1008037. doi: 10.1371/journal.pcbi.1008037 (PMC7467302; doi:10.1371/journal.pcbi.1008037)
Supplement: S1 Table — (DOCX) [file pcbi.1008037.s001.docx]

**SUPPORTING INFORMATION**

| Initial Bacterial | Host survival | | | | | |
| --- | --- | --- | --- | --- | --- | --- |
| Load (Vib 79) | 0h | 24h | 48h | 72h | 96h | 120h |
| 10^7^ CFU/mL | 1 | 0 | 0 | 0 | 0 | 0 |
| 10^5^ CFU/mL | 1 | 1 | 0 | 0 | 0 | 0 |
| 10^3^ CFU/mL | 1 | 1 | 0.95 | 0.35 | 0.1 | 0.05 |

**Table S1:** Host survival data from [38]. G. mellonella were infected with different bacterial loads of Vib 79: 10^3^ CFU/mL, 10^5^ CFU/mL and 10^7^ CFU/mL. Larvae were then monitored at the end of every 24 h period, up to 120 h, and their survival rates recorded. No antibiotic, or other treatments, were administered during this time.
