# Supplementary material for: Optimising efficacy of antibiotics against systemic infection by varying dosage quantities and times
Source: PLoS Comput Biol. 2020 Aug 3;16(8):e1008037. doi: 10.1371/journal.pcbi.1008037 (PMC7467302; doi:10.1371/journal.pcbi.1008037)
Supplement: S2 Table — (DOCX) [file pcbi.1008037.s002.docx]

**SUPPORTING INFORMATION**

| Time (h) | Concentration (mg/L) | Standard deviation |
| --- | --- | --- |
| 0 | 110 |  |
| 0.25 | 19.00 | 0.94 |
| 0.5 | 17.65 | 0.81 |
| 1 | 11.97 | 1.85 |
| 1.5 | 8.22 | 0.88 |
| 2 | 11.12 | 0.28 |
| 4 | 7.62 | 1.67 |
| 7.5 | 9.94 | 1.21 |
| 24 | 8.01 | 1.42 |
| 29 | 4.80 | 0.39 |

**Table S2:** Experimental data from the tetracycline half-life experiments. Mean concentration of TET over time, along with the standard deviation across the repeated experiments.
