# Supplementary material for: Optimising efficacy of antibiotics against systemic infection by varying dosage quantities and times
Source: PLoS Comput Biol. 2020 Aug 3;16(8):e1008037. doi: 10.1371/journal.pcbi.1008037 (PMC7467302; doi:10.1371/journal.pcbi.1008037)
Supplement: S3 Table — (DOCX) [file pcbi.1008037.s003.docx]

**SUPPORTING INFORMATION**

| **group** | **infected** | **treatment (mg/kg)** | | **relative survival** | | | | | | | |  |
| --- | --- | --- | --- | --- | --- | --- | --- | --- | --- | --- | --- | --- |
|  | 0 h | 2 h | 24 h | 24h | 48h | 72h | 96h | 120h | 144h | 168h | 192h | |
| unmanipulated | - | - | - | 90 | 90 | 90 | 90 | 90 | 90 | 90 | 90 | |
| PBS only | PBS | PBS | PBS | 90 | 90 | 90 | 90 | 90 | 90 | 90 | 89 | |
| antibiotics only | PBS | 0.9 | 0.34 | 60 | 60 | 60 | 60 | 60 | 60 | 60 | 60 | |
| positive control | Vib 79 | PBS | PBS | 90 | 0 | 0 | 0 | 0 | 0 | 0 | 0 | |
| 0.9 mg | Vib 79 | 0.56 | 0.34 | 90 | 88 | 78 | 69 | 48 | 28 | 21 | 19 | |
| 0.9 mg | Vib 79 | 0.9 | PBS | 90 | 88 | 82 | 79 | 59 | 43 | 31 | 22 | |
| unmanipulated | - | - | - | 90 | 90 | 90 | 90 | 90 | 90 | 90 | 88 | |
| PBS only | PBS | PBS | PBS | 90 | 90 | 90 | 90 | 90 | 90 | 90 | 89 | |
| antibiotics only | PBS | 0.76 | 0.7 | 90 | 90 | 90 | 90 | 89 | 89 | 89 | 88 | |
| positive control | Vib 79 | PBS | PBS | 88 | 0 | 0 | 0 | 0 | 0 | 0 | 0 | |
| 0.9 mg | Vib 79 | 0.76 | 0.14 | 90 | 90 | 86 | 63 | 54 | 42 | 31 | 23 | |
| 0.9 mg | Vib 79 | 0.45 | 0.45 | 90 | 90 | 76 | 46 | 36 | 27 | 19 | 9 | |
| 0.9 mg | Vib 79 | 0.2 | 0.7 | 90 | 86 | 45 | 11 | 2 | 1 | 1 | 0 | |

**Table S4**: Validation experiments. Full experimental data for host survival for various antibiotic treatments, along with control groups, used to validate the mathematical model. The experiments were done in two stages, with the top half and bottom half of the table representing the two stages.
